# Challenging diagnosis: unveiling extensive multisystemic sarcoidosis with musculoskeletal involvement through a clinically ambiguous soft tissue mass in the palm

**DOI:** 10.1007/s00256-024-04787-0

**Published:** 2024-09-17

**Authors:** Julius M. Weinrich, Lennart Well

**Affiliations:** 1 Diagnostic Radiology, Kernspinzentrum Hamburg, Hamburg, Germany; 2 Department of Diagnostic and Interventional Radiology and Nuclear Medicine, University Hospital Hamburg Eppendorf, Hamburg, Germany

**Keywords:** Sarcoidosis, Musculoskeletal involvement, MRI

## Abstract

We report about a 33-year-old man who was referred for assessment of a progressively enlarging mass of the palmar hand muscles, serving as the initial indication of extensive multisystemic sarcoidosis with musculoskeletal involvement. The case underscores the diagnostic challenges associated with the indolent course of sarcoidosis, highlighting the need for recognizing seemingly benign symptoms for early detection. Musculoskeletal imaging findings presented in the case stress the importance of considering sarcoidosis in the differential diagnosis of orthopedic cases. This report emphasizes the importance of understanding possible musculoskeletal imaging findings in sarcoidosis, thereby enabling radiologists to effectively guide patient management.

## Introduction

Sarcoidosis, a granulomatous disease affecting multiple organ systems, presents a diagnostic conundrum due to its capacity to mimic various pathologies [1]. Its etiology involves a complex interplay of genetic predisposition, particularly involving HLA factors, and T-cell-mediated immune responses triggered by an unidentified antigen. A common finding in sarcoidosis is bilateral symmetric hilar adenopathy on chest radiographs, which is part of a distinctive clinical manifestation of systemic sarcoidosis known as Löfgren syndrome. Patients with Löfgren syndrome typically present with fever, erythema nodosum, polyarticular arthritis, and bilateral hilar lymphadenopathy. Another possible clinical manifestation is Heerfordt-Waldenström syndrome, in which patients present with uveitis, parotid gland enlargement, fever, and facial nerve palsy. While elevated serum levels of angiotensin-converting enzyme (ACE) and vitamin D, in conjunction with non-caseating granulomas on biopsy, are suggestive, they lack specificity, mandating the exclusion of alternative diagnoses such as rheumatoid arthritis and fungal infections [2]. Despite asymptomatic muscle involvement being common in sarcoidosis, radiologically evident bone involvement is rare, particularly at initial presentation [1].

## Case report

**Fig. 1:**
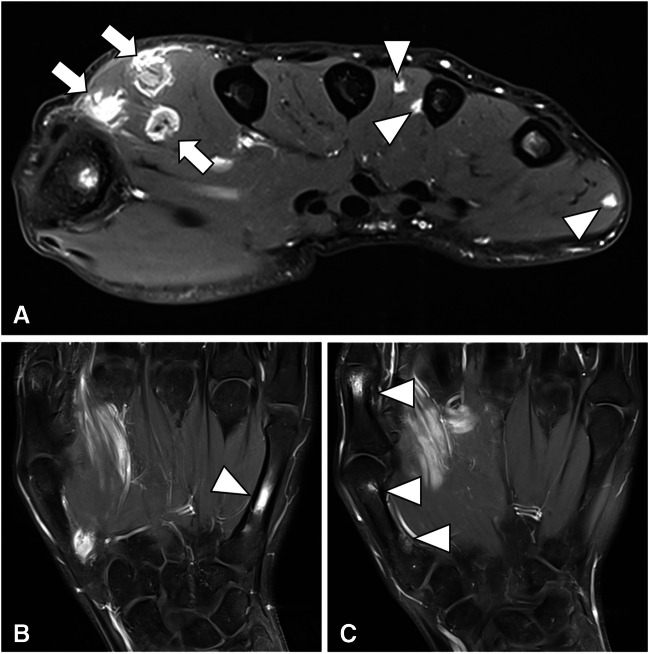
Contrast-enhanced T1-weighted MRI of the hand reveals several enhancing small (**A**, arrowheads) and medium-sized (**A**, arrows) nodular lesions of the dorsal hand muscles. Further, it depicts enhancing bone lesions in the first and fifth metacarpals (**C**, arrowhead). Simultaneous presence of bone and soft tissue lesions indicates a systemic disease, whereas the nodular shape is suggestive of a granulomatous disease

**Fig. 2:**
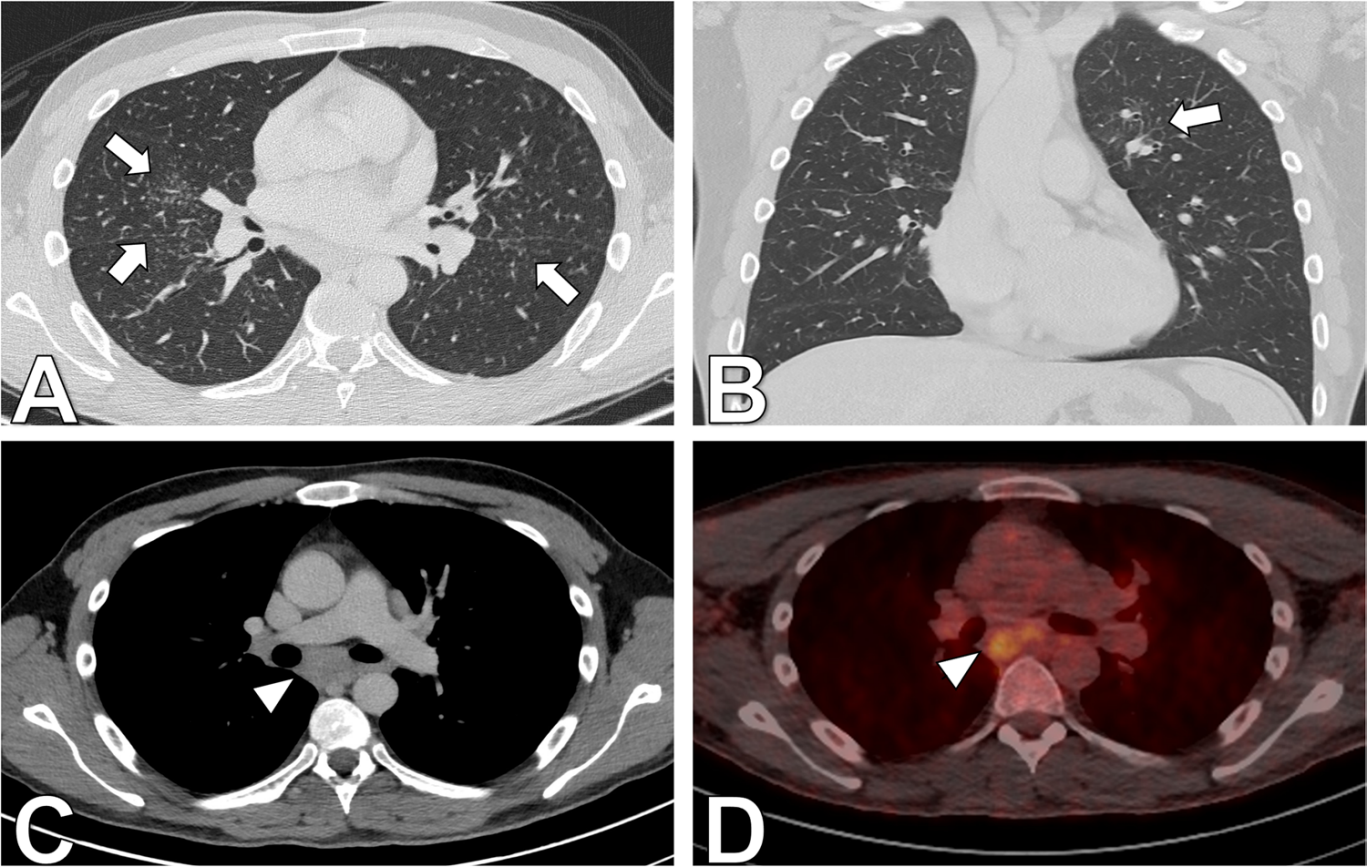
Contrast-enhanced 18-F-fluorodeoxyglucose positron emission computed tomography (PET/CT) with axial (**A**) and coronal (**B**) lung window showing fine reticulonodular opacities (arrows) of the lung parenchyma. Axial (**C**) soft tissue window of the mediastinum with enlarged mediastinal lymph nodes (arrowhead) and axial fused PET/CT of the mediastinum (**D**) with increased FDG uptake of enlarged mediastinal lymph nodes (arrowhead)

**Fig. 3:**
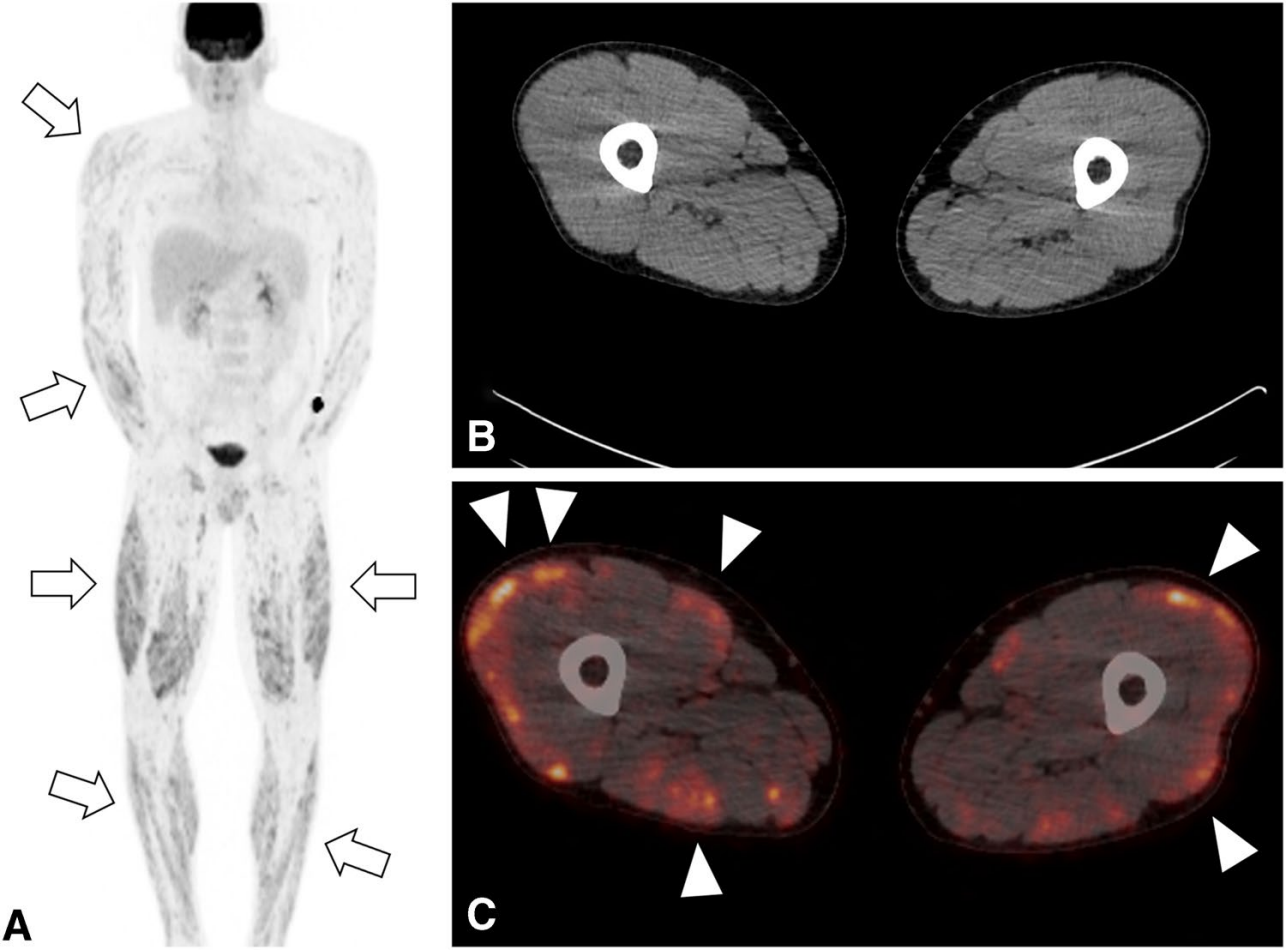
Coronal maximum intensity projection (MIP) of the 18-F-fluorodeoxyglucose positron emission computed tomography (PET/CT), **A** displaying increased metabolic activity of the muscles of the upper and lower extremities (arrows). Axial CT (**B**) and fused PET/CT (**C**) images of the thigh with nodular appearance of metabolic activity of the muscles (arrowheads) without a morphological correlate in the CT

Patient consent was obtained in accordance with IRB and institutional privacy requirements. This case report details the presentation of a 33-year-old man referred for evaluation of progressive enlargement of the palmar hand muscles, ultimately serving as the initial indication of extensive sarcoidosis with musculoskeletal involvement. The MRI revealed nodular space-occupying lesions within the dorsal interosseus muscles and abductor indicis muscle, along with localized bone lesions in the first and fifth fingers (Fig. 1). Suspicion of granulomatous diseases, such as sarcoidosis, arose, prompting further evaluation. Laboratory findings showed elevated levels of ACE, calcium, vitamin D, and glomerular filtration rate (GFR). Clinical examination revealed nodular changes in both forearms and the neck, painful swelling in both lower legs, and physical limitations initially attributed to a previous COVID infection. However, chest CT demonstrated pulmonary and nodal involvement of sarcoidosis Stage II (Fig. 2), confirmed by bronchial lavage exhibiting CD4-dominant lymphocytosis. Subsequent PET-CT highlighted FDG-avid lesions in the spleen, dorsal spine muscles, lower leg muscles, and hand/forearm as well as mediastinal lymph nodes (Fig. 3). Diagnosis was histopathologically confirmed via renal, muscle, and skin biopsies, revealing multisystemic sarcoidosis with cutaneous, muscular, osseous, and kidney involvement. Treatment involved initial administration of prednisolone followed by corticosteroid-sparing supplementation with azathioprine. The patient was discharged in good health after 22 days of inpatient treatment.

## Discussion

Sarcoidosis, a granulomatous disease of unknown origin, has confounded clinicians since its initial description in 1877 [3]. Often misdiagnosed as tuberculosis, sarcoidosis presents with non-caseating granulomas, preventing the identification of a specific causative agent despite exhaustive investigation. While musculoskeletal involvement is common, occurring in 1% to 50–83% of cases, histologically proven muscular and bony involvement often lacks specific symptoms (4,5). Notably, sarcoid myopathy may manifest in various forms, including nodular myopathy, chronic myopathy, or acute myositis, with nodular, tumor-like manifestations being rare [4]. Few case series document nodular muscular involvement in sarcoidosis, with even fewer reporting hand manifestations as the initial symptom [6–8]. This case contributes significantly to the literature by highlighting an unusual manifestation of sarcoidosis in the musculoskeletal system. Knowledge of imaging findings in musculoskeletal sarcoidosis manifestations facilitated timely diagnosis and treatment in this patient, underscoring the importance of awareness and recognition of diverse clinical presentations in sarcoidosis management.
